# The association between major dietary patterns and severe mental disorders symptoms among a large sample of adults living in central Iran: Baseline data of YaHS-TAMYZ cohort study

**DOI:** 10.1186/s12889-022-13518-w

**Published:** 2022-06-04

**Authors:** Shamim Shams-Rad, Reza Bidaki, Azadeh Nadjarzadeh, Amin Salehi-Abargouei, Barbora de Courten, Masoud Mirzaei

**Affiliations:** 1 Nutrition and Food Security Research Center, Shahid Sadoughi University of Medical Sciences, Yazd, PO Code 8915173160 Iran; 2 Department of Nutrition, School of Public Health, Shahid Sadoughi University of Medical Sciences, Yazd, Iran; 3 Research Center of Addiction and Behavioral Sciences, Diabetes Research Center, Department of Psychiatry, Faculty of Medicine, Shahid Sadoughi University of Medical Sciences, Yazd, Iran; 4 Department of Medicine, School of Clinical Sciences, Monash University, Melbourne, VIC Australia; 5 School of Health and Biomedical Sciences, RMIT University, Melbourne, VIC 3085 Australia; 6 Yazd Cardiovascular Research Centre, Non-communicable Research Institute, Shahid Sadoughi University of Medical Sciences, Yazd, Iran

**Keywords:** Dietary patterns, Severe Mental Disorders Symptoms, Depression, Anxiety, Stress

## Abstract

**Background:**

The diet’s role in developing psychological disorders has been considered by researchers in recent years.

**Objective:**

To examine the association between major dietary patterns and severe mental disorders symptoms in a large sample of adults living in Yazd city, central Iran.

**Methods:**

This cross-sectional study used the baseline data of a population-based cohort study (Yazd Health study: YaHS). Dietary intakes were assessed by a multiple-choice semi-quantitative food frequency questionnaire (FFQ, Yazd nutrition survey called TAMYZ). Psychological assessments were also done by using the depression, anxiety, and stress scale-21 (DASS-21) questionnaire. Major dietary patterns were identified using principal component analysis (PCA). Analysis of covariance (ANCOVA) and logistic regression analyses were used to evaluate the relationship between dietary patterns and mental disorders symptoms.

**Results:**

A total of 7574 adults were included in the current analysis. Four major dietary patterns were identified: "Sugar and Fats”, “Processed Meats and Fish”, "Fruits" and “Vegetables and Red Meat”. After adjustment for all confounding variables, participants in the fifth quintile of “Fruits” dietary pattern which was highly correlated with dried fruits, canned fruits, fruit juice, olive, hydrogenated fats and fruits intake, had a lower odds of severe depression (OR=0.61, 95% CI: 0.45–0.81, p for trend=0.057), anxiety (OR=0.64, 95% CI: 0.50–0.80, p for trend=0.007), and stress, (OR=0.45, 95% CI: 0.30–0.68, p for trend=0.081).

**Conclusions:**

The intake of a dietary pattern high in dried fruits, canned fruits, fruit juice, olive, hydrogenated fats, and fruits might be inversely associated with depression, anxiety, and stress symptoms. Future prospective studies are needed to warrant this finding.

**Supplementary Information:**

The online version contains supplementary material available at 10.1186/s12889-022-13518-w.

## Background

Mental disorders are diseases that affect emotion, cognition, and behavioral control and affect almost 30% of people across the lifespan [1, 2]. A large number of people are affected by common mental disorders including depression and anxiety around the world [3]; between 1990 and 2013, the number of individuals suffering from depression and/or anxiety increased by almost 50%, from 416 million to 615 million [4]. Furthermore, depression, anxiety, and psychological distress are regarded as the important causes for disability, high economic burden, and early mortality [5]. It has been shown that depression and anxiety are prevalent among 21% and 20.8% of Iranians, respectively which may be underestimated because of the stigma these diseases are associated with [6].

There are different factors influencing people’s mental health including quality of life, demographic and financial factors, type and severity of current stressors, physical disorders, history of trauma, etc. [7, 8]. Furthermore, It is proposed that lifestyle changes might explain the increased prevalence of mental disorders over recent decades [9]. Dietary intakes of foods and beverages are also considered as a potentially modifiable factor involved in the etiology of mental disorders [10]. The majority of previous investigations regarding the association between diet and mental disorders have focused on individual nutrients, specific foods, and food groups [11]. For example, dietary intakes of iron [12], selenium and zinc [13], vitamin B6 [14], folate, vitamin B12 [13], omega-3 fatty acids [15], choline [16], fish [17], and vegetables [18] are associated with depression, anxiety, and stress. However, foods are not usually consumed individually. So their combined effect on mental disorders may differ from their isolated effects [19].

Empirically derived dietary patterns have lately appeared in nutritional epidemiology to examine associations between diet and chronic diseases [20]. In this approach, multiple nutrients or foods are combined using statistical methods to derive a single variable, namely dietary pattern [21]. It has been supposed that dietary patterns provide a better and more general look into diet-disease relations [20] and may be more predictive of chronic disease risk than individual foods or nutrients [21].

Several studies have assessed the association between empirically derived dietary patterns and mental disorders. For instance, a study on Australian adult women showed that a "traditional" dietary pattern (high intakes of fruit, vegetables, whole grains, meat, and fish) was associated with lower odds of major depression and anxiety disorders [22]. In addition, adherence to a "whole food" dietary pattern was linked with decreased risk, while a "processed food" dietary pattern increased the risk of depression in middle-aged British women [23]. Also, a dietary pattern high in fruits, vegetables, mushrooms, seaweed, potatoes, soybean products, and fish/shellfish, named “healthy Japanese” dietary pattern, was inversely associated with depressive symptoms among Japanese women [24]. A study of middle-aged adults in eastern China indicated that a “grains-vegetables dietary pattern” (high consumption of whole grains, fresh fruit, fresh vegetables, tuber, miscellaneous bean, and honey) is associated with a decreased risk, and a western dietary pattern (high consumption of processed meat, red meat, seafood, freshwater fish and shrimp, dairy products, nuts, snacks, fats, fast foods, desserts, soft drinks, and coffee) is linked with an increased risk of anxiety [25]. In the Norwegian population, a western-type diet was associated with increased anxiety in women and men before final adjustment for energy intake; furthermore, a “traditional Norwegian dietary pattern” was also linked with reduced depression in women and anxiety in men [26]. Similar findings have also been demonstrated in Chinese adolescents [27]. In line with these findings, a strong positive association has been found between the western dietary pattern and anxiety and stress; also, there was an inverse association between a Mediterranean-type dietary pattern and anxiety in an Iranian population [28]. The majority of studies have tried to assess the relationship between dietary patterns and depression, while a few studies have focused on the association between dietary patterns and anxiety [29].

It is worth mentioning that the relationship between dietary patterns and mental health is complex and may be bidirectional [30]. For instance, some changes in food choices are prompted by depressive symptoms; diminished appetite is a symptom of major depression for many people and there is also evidence to suggest that some people with depressive symptoms are more likely to consume more fat and sugars [31] as well as fewer fruits and vegetables [32].

The previous studies from the Middle East were conducted with a limited number of participants and led to inconsistent results; furthermore, the major dietary patterns might be different between societies with heterogeneity in food culture, like Iran [33, 34]. Therefore, the present study aimed to examine the association between major dietary patterns identified by principal components analysis and depression, anxiety, and stress symptoms in a large sample of adults living in Yazd city in central Iran.

## Methods

### Study setting and population

The present study was a cross-sectional study carried out on the recruitment phase data of a population-based cohort study entitled: “Yazd Health Study (YaHS)”, which has been the most comprehensive study on the health and diseases in Yazd greater area (www.yahs-ziba.com). About 10000 inhabitants of Yazd city were selected using a two-level clustered random sampling method according to WHO STEP guidelines. The 200 clusters were selected randomly according to city postcodes, and 50 participants were assigned to each cluster (25 men and 25 women; five persons in each 10-year age group, e.g. 20–29, 30–39, 40–49, 50–59 and 60–69 years).

### Study design

The detailed information on the study design, participants recruitment, and data collection methods are explained previously [35]. In the YaHS study, data on general characteristics, personal and dietary habits, physical activity, medical history, mental health status, and social well-being of the participants plus blood pressure, and anthropometric measurements were collected from 10000 participants by trained interviewers (November 2014-April 2016). Meanwhile, in the second phase (December 2015), data on dietary foods and supplements intake were collected from all participants entered into YaHS study, in a study named as Yazd Nutrition Survey (YNS) which is locally known as TAMYZ in Persian (TAghzieh-e-Mardome YaZd) by trained interviewers using a multiple-choice semi-quantitative food frequency questionnaire (FFQ). A unique code was assigned to each participant in the YaHS study and the same code was used to enter dietary intakes data in the TAMYZ study. The code was used to merge the collected data. After merging data from YaHS and TAMYZ, 9962 participants were left for further analysis. Participants with missing data on DASS-21 questionnaire and dietary intakes (*n*=1029), and those with chronic diseases including heart disease, and different cancers (*n*=909) were removed. In addition, those with energy intake lower than 800 Kcal and higher than 7000 Kcal were considered as under- and over-reporters, respectively, and were removed from the study. Overall, 7574 participants had complete data and were entered into the current analysis. In YaHS and TAMYZ written informed consents for entering the study and publication of study results were taken from all participants. The methodology of the present study was also approved by the ethics committee of Shahid Sadoughi University of Medical Sciences (approval code: IR.SSU.SPH.REC.1398.011).

### Dietary assessment method

The dietary assessment in TAMYZ was done by using a 178-item semi-quantitative multiple-choice FFQ [36]. For each food item, participants were asked to report the i) frequency of food consumption in the past year based on 10 multiple-choice frequency response categories varying from ‘never or less than once a month’ to ‘10 or more times per day, and ii) amount of food consumed each time (portion size). The portion size was determined using questions with five predefined answer categories which were different, according to each food item. In a previous investigation, the median intraclass correlation between FFQs which were introduced 3 times to the same participants was 0.56. The median de-attenuated, age, sex, and education adjusted partial correlation coefficients for validity was 0.26 for weighted dietary food records (WDRs) and FFQ. Furthermore, the FFQ validity coefficients for vitamin C, calcium, magnesium, and zinc were 0.13, 0.62, 0.89, and 0.66, respectively, using the triads method. The median exact agreement and complete disagreement between FFQ and WDRs were 33% and 6%, respectively. It was shown that the FFQ used in the current study is a reproducible and valid tool to assess the long-term dietary intake for large-scale studies in this population [36].

Furthermore, participants were asked to complete a separate multiple-choice questionnaire about the frequency of the selected supplements (ie, vitamin D, calcium, iron, folic acid, fish oil (or omega-3), and multivitamin-mineral supplements). All reported intakes were converted to g/day by using household portion sizes of consumed foods [37]. The USDA food database was used to calculate nutrient intakes [38]. A total of 40 food groups were constructed by summing up the food items according to the similarities in their nutrient profiles and culinary usage (Supplementary Table 1), and the food groups were used to identify dietary patterns.

### Assessment of the psychological profile

The depression, anxiety, and stress Scale -21 (DASS-21) questionnaire was used to assess depression, anxiety, and stress symptoms. This questionnaire was validated by Sahebi et al. for the Iranian population. The correlation between the Depression subscale and the Beck Depression Inventory scale was +0.70, between the Anxiety subscale and Zung Anxiety Inventory was +0.67, and between the Stress subscale and Perceived Stress Inventory was +0.49 and all correlations were statistically significant [39]. The questionnaire is composed of three 7-item subscales: depression, anxiety, and stress. Participants were asked to rate how much each item described their experience over the past week ranging from 0 (did not apply to me at all – never) to 3 (applied to me very much, or most of the time–almost always). Subscale scores were calculated by summing up the related items. Therefore, participants’ DASS-21 score for each subscale ranged from 0 to 21. Generally, higher scores indicate a greater level of psychological disorders. Participants were classified into one of the five primary classifications based on their scores, which include the absence of disease, mild, moderate, severe, and very severe [39–41]. Finally, the individuals were classified into two main categories: “absence of disease, mild, and moderate psychological disorders symptoms” and “with severe psychological disorders symptoms” (individuals who were classified as severe and very severe). The classification of symptoms for each mental disorder was done based on a method proposed by Sahebi et al. (Table 1) [39].

**Table 1:** Cut-off points used for classification of mental disorders’ symptoms severity using depression, anxiety, and stress Scale -21 (DASS-21) questionnaire [39]

Classifications	Depression score		Anxiety score		Stress score	
	Males	Females	Males	Females	Males	Females
Absence of disease, Mild and moderate	0-12	0-14	0-11	0-12	0-15	0-17
Severe and very severe	≥13	≥15	≥12	≥13	≥16	≥18

### Anthropometric measurements

Anthropometric measurements including height, weight, waist circumference, and hip circumference were performed three times (before starting the interview, again after completing one-third of the questionnaire, and for a final time after having completed two-thirds of the questionnaire) by trained interviewers. The average of these three measurements was considered as the final measure. Also, BMI was calculated as weight (kg) divided by height squared (m).

### Assessment of other variables

Demographics including age, gender, marital status (single/married/divorced or widow), education (uneducated/middle school/high school/bachelor’s degree/master’s degree or higher), job status (unemployed/government-employed/manual worker/self-employed), smoking status (never smoker/current smoker/ex-smoker), diabetes (yes/no), hypertension (yes/no), and homeownership status (yes/no) were collected through a self-administered questionnaire. The short version of the International Physical Activity Questionnaire (IPAQ) was used to measure physical activity level and results were expressed as metabolic equivalent in minutes per week (MET-min/wk) [42].

### Statistical analysis

Principal components analysis with orthogonal transformation was used to derive major dietary patterns based on forty food groups and the factors were rotated by using varimax rotation. Eigenvalues (>1), scree plot, and factor interpretability were considered to select the major dietary patterns [43]. Each food group received a factor loading associated with each dietary pattern. Factor loadings show the correlation coefficient between the food group and the dietary pattern. In the current study, food groups with factor loadings of more than 0.3 were thought to be strongly associated with the factors, and were considered as the most informative variable for describing the dietary patterns. Labels were given to different dietary patterns, even though these did not perfectly describe each underlying pattern. After that, the factor score for each dietary pattern was computed by summing up intakes of food groups weighted by their factor loadings. Participants received a factor score for each identified dietary pattern and were categorized into quintiles (five groups with equal sample size) of dietary patterns’ scores. Participants in the lowest quintile (Q1) had the lowest adherence to the identified dietary pattern and those in the highest quintile (Q5) had the highest adherence to that dietary pattern.

The normal distribution of continuous variables was assessed using histogram and Kolmogorov-Smirnov test. Continuous (dietary nutrients intake, mental disorder scores, body weight, body mass index, waist circumference, hip circumference, and physical activity) and categorical variables (age group, sex, marital status, education, job status, smoking status, and homeownership) were compared across quintiles of dietary patterns intake scores using analysis of variance (ANOVA) and chi-square tests, respectively. We compared age, sex, and energy standardized dietary food groups and nutrients intakes across quintiles of dietary patterns' scores using analysis of covariance (ANCOVA) with Bonferroni correction. This method was also applied to compare depression, anxiety, and stress scores (as outcome variables) across quintiles of derived dietary patterns (as predictor variables) in crude and two multi-variable adjusted models. Age, sex (male/female) and energy intake (kcal/day) were adjusted in the first model (model 1), and then BMI (kg/m^2^), physical activity (MET-min/week), marital status (single/married/widowed or divorced), smoking status (yes/no), job status (unemployed /government-employed/manual worker/self-employed), education status (uneducated /middle school /high school or diploma /bachelor’s degree /master’s degree or higher), homeownership (yes/no), diabetes (yes/no) and hypertension (yes/no) were further adjusted in the second model (model 2). Furthermore, to determine the association between dietary patterns (as predictor variables), and the likelihood of developing depression, anxiety, and stress (as outcome variables), the binary logistic regression was applied in crude and multivariable-adjusted models. The overall trend of odds ratios across increasing quintiles of dietary pattern scores (p *for trend*), was examined by treating the quintile categories as an ordinal variable in the analyses. All statistical analyses were conducted using the Statistical Package for Social Sciences (SPSS, version 15.0 for Windows, 2006, SPSS, Inc, Chicago, IL). A p-value less than 0.05 was regarded as statistically significant.

## Results

### Dietary patterns

In total, 7574 participants (3763 males and 3811 females) were included in the current analysis. Four major dietary patterns were identified using principal components analysis, and they were labeled as “Sugar and Fats”, “Processed Meats and Fish”, “Fruits” and “Vegetables and Red Meat”. These four dietary patterns explained 18.63% of the total variation in dietary intakes in this population. The “Sugar and Fats” dietary pattern was characterized by high consumption of sweets and desserts, nuts, snack foods, broth, condiments, sugars, and mayonnaise and explained 6.87 % of the total variance. The “Processed Meats and Fish” dietary pattern was mainly loaded with processed meats, fish, and organ meats and explained by 4.12 % of the total variance. The "Fruits" dietary pattern was associated with higher intakes of dried fruits, canned fruits, fruit juice, olive, hydrogenated fats, and fruits and explained 3.86% of the total variance. Tomatoes, green leafy vegetables, other vegetables, red meat, and fruits were highly loaded in the “Vegetables and Red Meat” dietary pattern which was explained by 3.78 % of the total variance. All food groups as well as their loading factors for each dietary pattern are shown in Table 2. The high positive loadings demonstrate strong positive relation between food groups and dietary patterns, whereas high negative loadings indicate a strong negative association.

**Table 2:** Loading factor for foods and food groups based on major dietary patterns derived from principal component analysis^a^

	Factor 1	Factor 2	Factor 3	Factor 4
Sweets and desserts	0.672	-	-	-
Nuts	0.604	-	-	-
Soft drink	0.585	-	-	-
Snacks	0.532	-	-	-
Broth	0.531	-	-	-
Condiment	0.512	-	-	-
Sugars	0.489	-	-	-
Mayonnaise	0.444	-	-	-
Processed meats	-	0.579	-	-
Fish	-	0.520	-	-
Organ meats	-	0.505	-	-
Yoghurt drink	-	-	-	-
Dried fruits	-	-	0.604	-
Canned fruits	-	-	0.580	-
Fruit juice	-	-	0.491	-
Olive	-	-	0.376	-
Tomatoes	-	-	-	0.648
Green leafy vegetables	-	-	-	0.486
Other vegetables	-	-	-	0.456
Fruits	-	-	0.30	0.364
Potatoes	-	-	-	-
French fries	-	-	-	-
Red meats	-	-	-	0.425
Refines grain	-	-	-	-
Vegetable oils	-	-	-	-
Low-fat dairy products	-	-	-	-
Salt	-	-	-	-
Eggs	-	-	-	-
Cruciferous vegetables	-	-	-	-
Poultry	-	-	-	-
Butter	-	-	-	-
Margarine	-	-	-	-
Pickles	-	-	-	-
Tea	-	-	-	-
Legumes	-	-	-	-
Coffee	-	-	-	-
Hydrogenated fats	-	-	0.314	-
High-fat dairy products	-	-	-	-
Whole grain	-	-	-	-
Yellow vegetables	-	-	-	-
**Total variation explained**	**6.87**	**4.12**	**3.86**	**3.78**

^a^Loading factors lower than 0.3 are not shown for better interpretation of major dietary patterns

### Participants’ characteristics

The general characteristics of the study participants across quintiles of dietary patterns’ (DPs’) scores are presented in Table 3. Participants in the fifth quintile of the “Sugar and Fats” pattern were more likely to be younger, employed, with higher physical activity, with low education, and with lower waist and hip circumferences (p<0.05). Participants with the highest “Processed meats and Fish” dietary pattern score were younger, with higher physical activity, and with lower waist circumference (p<0.05). Participants in the top quintile of the “Fruits” dietary pattern had a higher body mass index, waist and hip circumferences, lower physical activity, average education (high school diploma). The adherence to the “Vegetables and Red Meat” diet was associated with average education (high school diploma). There was no significant difference in other quantitative and qualitative variables across quintiles of the “Vegetables and Red Meat” dietary pattern (Table 3).

**Table 3:** General characteristics of study participants according to quintiles of major dietary patterns’ score

	Sugar and Fats dietary pattern			Processed meat and Fish dietary pattern			Fruits dietary pattern			Vegetables and Red Meat dietary pattern			Total population
	Q1	Q5	***p*** Value	Q1	Q5	***p*** Value	Q1	Q5	***p*** Value	Q1	Q5	***p*** Value	
**Body weight (kg)**	72.51±14.17	73.40±15.03	0.39	72.82±14.17	72.93±14.79	0.61	72.62±14.55	72.78±14.89	0.95	72.01±14.57	73.44±14.47	0.11	72.74±1.45
**Body mass index (kg/m2)**	27.16±5.11	27.16±5.27	0.14	27.27±5.21	26.95±5.12	0.15	26.77±5.15	26.95±5.25	0.04	26.85±5.14	27.19±5.02	0.34	27.00±5.17
**Waist circumference (cm)**	94.66±12.97	94.39±13.04	0.00	94.55±12.82	92.78±13.43	0.00	92.97±13.04	92.99±13.13	0.01	93.23±13.26	94.00±13.34	0.42	93.60±1.32
**Hip circumference (cm)**	103.28±10.55	100.88±12.07	0.00	102.10±11.07	101.22±11.82	0.26	100.97±11.77	101.59±11.34	0.02	101.40±11.62	102.42±11.35	0.06	101.74±11.48
**Physical activity (MET-min/week)**	831.22±866.15	911.47±912.93	0.00	840.88±880.24	968.66±936.27	0.00	981.80±949.77	909.97±905.53	0.00	884.12±921.41	931.80±926.61	0.55	901.16±905.16
**Depression score**	3.50±3.97	3.41±3.69	0.08	3.50±3.86	3.42±3.86	0.10	3.60±4.07	2.99±3.47	0.00	3.53±3.91	3.49±3.75	0.01	3.33±3.79
**Anxiety score**	3.33±3.83	2.81±3.37	0.00	3.11±3.67	2.74±3.51	0.04	3.22±3.88	2.70±3.48	0.00	3.15±3.76	3.18±3.71	0.01	2.99±3.65
**Stress score**	5.89±4.85	5.91±4.53	0.73	5.94±4.64	6.29±4.83	0.01	6.14±4.84	5.97±4.71	0.09	5.96±4.81	6.19±4.69	0.04	5.93±4.70
**Age group (%)**													
20-29	18.8	22.5	0.00	19.4	23.4	0.01	23.1	21.8	0.07	21.1	21.3	0.56	20.9
30-39	19.5	23.5		21.0	23.1		21.2	23.7		22.0	21.7		21.1
40-49	21.6	21.6		22.0	21.6		21.5	21.9		21.6	21.3		20.9
50-59	20.9	17.3		19.5	18.0		19.7	18.2		18.3	20.6		18.5
60-69	19.2	15.1		18.2	13.9		14.5	14.4		17.1	15.0		16.0
**Sex (female) (%)**	52.2	49.6	0.40	51.9	48.9	0.39	47.8	49.5	0.19	49.9	49.8	0.62	49.0
**Marital status (%)**													
Single	10.7	11.6	0.36	11.2	11.8	0.99	11.9	12.8	0.62	12.4	11.9	0.46	11.3
Married	85.8	85.6		85.5	84.9		84.8	84.2		85.2	84.9		82.7
Widowed or divorced	3.5	2.8		3.2	3.3		3.3	3.0		2.4	3.3		3.2
**Education (%)**													
Uneducated	25.5	21.4	0.00	23.4	23.6	0.57	22.7	24.3	0.00	25.2	23.3	0.00	23.4
Middle school	28.8	32.0		30.6	26.8		32.0	25.6		28.8	27.5		27.7
High School	31.3	30.1		30.5	30.5		30.2	33.3		30.4	32.3		30.2
Bachelor’s degree	12.2	13.9		12.8	12.8		11.6	13.3		13.9	12.7		13.1
Master’s degree or higher	2.2	2.6		2.6	2.6		3.5	3.5		1.7	4.3		2.7
**Job status (%)**													
Unemployed	18.4	22.6	0.00	20.8	19.5	0.27	20.4	18.5	0.25	20.2	20.6	0.62	18.7
Government-employed	52.5	45.4		49.4	46.1		46.4	48.2		48.6	46.4		46.7
Manual worker	3.3	4.1		3.3	3.6		3.4	3.5		3.5	3.1		3.3
Self-employed	25.7	28.0		26.6	30.8		29.7	29.8		27.6	29.8		27.0
**Smoking status (%)**													
Never smoker	88.1	88.0	0.89	87.0	89.3	0.10	86.0	89.0	0.14	87.4	86.8	0.78	83.5
Current smoker	10.8	10.4		10.9	9.6		12.2	9.9		11.0	11.4		10.1
Ex-smoker	1.1	1.6		2.1	1.1		1.9	1.1		1.5	1.8		1.5
**Home ownership (%)**													
Yes	21.7	22.5	0.12	22.2	21.3	0.84	23.8	20.4	0.02	23.4	20.5	0.21	20.8
No	78.3	77.5		77.8	78.7		76.2	79.6		76.6	79.5		76.1

^1^Values are reported as Mean ± Standard Deviation (SD) otherwise indicated

^2^The quantitative and qualitative variables were compared across quintiles of dietary patterns’ scores using the analysis of variance and Chi-square tests, respectively

### Dietary food and nutrients intakes

Age-, sex- and energy-adjusted intakes of selected food groups and nutrients across quintile categories of major DPs’ scores are provided in Table 4. Compared with those in the lowest quintile of the “Sugar and Fats” dietary pattern, participants in the top quintile had significantly higher intakes of energy, total carbohydrate, mono-unsaturated, poly-unsaturated and total fat, sugar, vitamin E (alpha-tocopherol), and nuts intake (*p* < 0.05); however, they had lower intakes of whole and refined grains, low and high-fat dairy products, processed and red meats, legumes, fruits, vegetables, total protein, saturated fat, vitamin C, thiamine, riboflavin, vitamin B6, B12, folic acid, magnesium, calcium, and iron (*p* < 0.05). Participants in the highest quintile of the “Processed Meats and Fish” dietary pattern had significantly higher intakes of refined grains, high-fat dairy products, processed meats, vegetables, legumes, energy, saturated, mono-unsaturated and total fat, total protein, thiamine, riboflavin, vitamin B6, B12, folic acid, magnesium, and calcium (*P* < 0.05). Individuals in higher quintiles of the “Fruits” dietary pattern consumed more refined grains, low-fat dairy products, fruits, vegetables, energy, total protein, vitamins C, E (alpha-tocopherol), thiamine, riboflavin, B6, B12, folic acid, magnesium, calcium, and iron (*p* < 0.05). Furthermore, subjects in the highest quintiles consumed fewer amounts of high-fat dairy products, legumes, nuts, red meat, total carbohydrate, saturated, mono-unsaturated, and total fat (*p* < 0.05). The “Vegetables and Read Meat” dietary pattern was positively associated with high-fat dairy products, legumes, fruits, vegetables, red meat, energy, total protein, vitamin C, E (alpha-tocopherol), thiamine, riboflavin, vitamin B6, B12, folic acid, magnesium, calcium, and iron intake and inversely associated with whole and refined grains, low-fat dairy products, nuts, processed meats, saturated, poly-unsaturated and total fat and total carbohydrate intake (*p* < 0.05).

**Table 4:** Comparison of age, sex and energy adjusted dietary food groups and nutrients intake according to quintiles of dietary food patterns

	Sugar and Fats dietary pattern			Processed meat and Fish dietary pattern			Fruits dietary pattern			Vegetables and Red Meat dietary pattern		
	Q1	Q5	*p* value	Q1	Q5	p value	Q1	Q5	p value	Q1	Q5	*p* value
**Food groups**												
**Whole grains (g/day)**	90.37±1.90	37.63±2.21	<0.001	97.70±1.83	55.81±2.04	<0.001	79.85±1.88	63.14±2.01	<0.001	95.84±1.86	68.55±1.93	<0.001
**Refined grains (g/day)**	306.53±4.46	73.29±5.18	<0.001	183.08±4.58	234.01±5.10	<0.001	211.22±4.64	217.09±4.95	0.03	283.75±4.55	211.52±4.72	<0.001
**Low fat dairy products (g/day)**	52.85±3.16	50.54±3.67	<0.001	77.34±3.04	37.66±3.38	<0.001	34.24±3.04	87.26±3.24	0.00	68.37±3.06	63.62±3.18	<0.001
**High fat dairy products (g/day)**	205.13±4.85	124.57±5.62	<0.001	141.86±4.64	247.84±5.16	<0.001	197.15±4.75	176.30±5.06	0.01	133.28±4.63	246.15±4.81	<0.001
**Nuts (g/day)**	12.09±0.88	51.63±1.02	<0.001	26.10±0.89	13.09±0.99	<0.001	23.39±0.90	15.51±0.96	<0.001	25.25±0.89	14.56±0.93	<0.001
**Legumes (g/day)**	52.53±1.63	35.93±1.89	<0.001	33.67±1.54	76.63±1.71	<0.001	69.00±1.56	40.80±1.67	<0.001	34.56±1.56	64.90±1.62	<0.001
**Processed meats (g/day)**	20.80±1.02	3.86±1.18	<0.001	6.13±0.94	40.48±1.05	<0.001	15.74±0.99	13.40±1.06	<0.001	23.46±0.98	8.29±1.02	<0.001
**Red meat (g/day)**	44.93±1.60	44.25±1.85	<0.001	65.31±1.54	35.40±1.72	<0.001	78.13±1.54	45.58±1.65	<0.001	30.60±1.44	99.56±1.50	<0.001
**Fruits (g/day)**	707.00±11.94	180.94±13.85	<0.001	604.56±11.91	346.87±13.26	<0.001	302.88±11.61	726.14±12.38	<0.001	303.53±11.27	800.44±11.72	<0.001
**Vegetables (g/day)**	302.11±6.89	187.65±7.99	<0.001	299.75±6.73	337.21±7.49	<0.001	304.94±6.78	327.17±7.23	<0.001	161.02±5.73	563.46±5.95	<0.001
**Nutrients**												
**Total energy**^**2**^ **(Kcal/day)**	2289.21±27.94	4482.36±27.81	<0.001	2825.11±30.81	4140.07±31.09	<0.001	2979.57±32.07	3969.11±31.86	<0.001	3046.58±33.51	3710.97.58±33.56	<0.001
**Total fat (g/day)**	97.61±0.95	137.03±1.10	<0.001	113.95±0.96	116.26±1.06	<0.001	116.69±0.96	107.76±1.03	<0.001	109.80±0.96	107.00±1.00	<0.001
**Saturated fat (g/day)**	30.50±0.30	28.80±0.35	<0.001	30.63±0.29	32.55±0.32	<0.001	33.05±0.29	30.09±0.31	<0.001	29.60±0.29	33.83±0.30	<0.001
**Mono-unsaturated fat (g/day)**	29.80±0.38	41.26±0.44	<0.001	36.21±0.37	36.58±0.42	<0.001	36.23±0.38	33.93±0.40	<0.001	33.75±0.38	33.35±0.39	0.08
**Poly-unsaturated fat (g/day)**	23.24±0.48	39.64±0.56	<0.001	31.93±0.48	25.16±0.53	<0.001	28.05±0.48	28.33±0.52	0.23	29.32±0.48	26.67±0.50	<0.001
**Total protein (g/day)**	123.63±0.96	78.11±1.11	<0.001	103.21±0.96	137.43±1.07	<0.001	115.38±1.00	121.05±1.07	<0.001	115.63±0.99	123.57±1.03	<0.001
**Total carbohydrate (g/day)**	416.33±2.42	425.48±2.81	<0.001	402.75±2.35	399.64±2.62	<0.001	400.42±2.36	397.02±2.52	<0.001	405.93±2.35	391.88±2.44	<0.001
**Simple sugar (g/day)**	19.24±1.04	63.69±1.20	<0.001	34.84±1.04	25.66±1.16	<0.001	52.92±1.01	18.05±1.08	<0.001	35.16±1.04	27.68±1.08	<0.001
**Vitamin C (μm /d)**	270.59±4.40	101.05±5.10	<0.001	249.20±4.38	185.70±4.87	<0.001	145.65±4.15	328.40±4.43	<0.001	148.87±4.14	322.08±4.30	<0.001
**Vitamin E (μm /d)**	21.98±0.82	28.65±0.95	<0.001	26.79±0.79	19.68±0.88	<0.001	19.50±0.79	26.82±0.85	<0.001	18.85±0.79	29.48±0.82	<0.001
**Thiamine (μm/d)**	2.58±0.01	1.45±0.01	<0.001	2.12±0.01	2.41±0.02	<0.001	2.16±0.01	2.29±0.01	<0.001	2.21±0.01	2.54±0.01	<0.001
**Riboflavin (μm/d)**	2.48±0.01	1.88±0.02	<0.001	2.17±0.01	2.74±0.02	<0.001	2.42±0.01	2.42±0.02	<0.001	2.19±0.01	2.76±0.01	<0.001
**Vitamin B6 (μm/d)**	2.58±0.02	2.47±0.03	0.03	2.45±0.02	2.68±0.02	<0.001	2.36±0.02	2.69±0.02	<0.001	2.32±0.02	2.88±0.02	<0.001
**Folic Acid (μm/d)**	407.79±4.27	309.76±4.95	<0.001	381.01±4.18	389.22±4.65	<0.001	364.65±4.18	418.47±4.46	<0.001	318.62±3.91	490.02±4.06	<0.001
**Vitamin B12 (μm/d)**	6.73±0.15	3.25±0.17	<0.001	4.86±0.14	9.29±0.16	<0.001	5.98±0.14	6.91±0.15	<0.001	5.68±0.14	7.19±0.15	<0.001
**Magnesium (mg/day)**	364.56±2.55	279.82±2.96	<0.001	331.10±2.52	364.24±2.81	<0.001	315.68±2.50	376.36±2.67	<0.001	307.03±2.39	402.45±2.49	<0.001
**Calcium (mg/day)**	1011.01±9.02	755.45±10.45	<0.001	911.54±8.93	1035.37±9.94	<0.001	959.18±9.03	994.37±9.64	<0.001	862.64±8.57	1170.15±8.90	<0.001
**Iron (mg/day)**	46.00±2.19	32.24±2.55	<0.001	45.62±2.12	44.32±2.36	0.41	36.79±2.13	54.27±2.27	<0.001	37.45±2.12	48.13±2.21	<0.001

^1^Values are reported as Mean ± Standard Error (SE)

^2^Values are adjusted for age and sex

### Comparison of mental disorders’ scores according to dietary patterns quintiles

Table 5 displays the crude and multivariable-adjusted mean scores for depression, anxiety, and stress across quintiles of dietary pattern scores. The analyses revealed that participants in the top quintile of the “Sugar and Fats” dietary pattern had a lower anxiety score than those in the bottom quintile in the crude model (crude: 2.81±0.09 vs. 3.33±0.09, *p* <0.001). The association remained significant even after adjustment for all possible confounds in model 2 (2.94±0.11 vs. 3.05±0.10, *p* = 0.01). We found no significant difference in depression and stress scores across quintiles of “Sugar and Fats” dietary pattern scores either in crude or multi-variable adjusted models. Although significant differences were observed in anxiety and stress scores between participants in different quintiles of “Processed Meats and Fish” dietary pattern in the crude model (*p* <0.05), the significant differences vanished after adjustment for all possible confounders (*p* >0.05). Participants who highly adhered to the "Fruits" dietary pattern had lower depression and anxiety scores compared to those with lower adherence to this DP (*p* <0.001) and the association remained significant after further adjustments for potential confounders in models 1 and 2 (*p* ≤ 0.05); There was no significant association between ‘Fruits’ dietary pattern and stress scores either in crude or multi-variable adjusted models (*p* > 0.05). Participants in the top quintile of “Vegetables and Red Meat” dietary had significantly higher depression, anxiety, and psychological distress scores either in crude or in multivariable-adjusted models (p < 0.05).

**Table 5:** Comparison of depression, anxiety and stress score according to quintiles of dietary food patterns in crude and multivariable adjusted models

	Depression score			Anxiety score			Stress score		
	Crude	Model 1^**2**^	Model 2^**3**^	Crude	Model 1	Model 2	Crude	Model 1	Model 2
**Factor 1: Sugar and Fats**									
Q1	3.50±0.10	3.44±0.10	3.33±0.10	3.33±0.09	3.23±0.09	3.05±0.10	5.89±0.12	5.92±0.12	5.86±0.13
Q2	3.36±0.10	3.31±0.10	3.28±0.10	3.06±0.09	2.94±0.09	2.91±0.10	5.81±0.12	5.86±0.12	5.84±0.13
Q3	3.14±0.10	3.13±0.10	3.08±0.10	2.72±0.09	2.68±0.09	2.62±0.09	5.99±0.12	6.04±0.12	6.03±0.13
Q4	3.24±0.10	3.26±0.10	3.25±0.10	3.05±0.09	3.09±0.09	3.05±0.09	6.04±0.12	6.02±0.12	6.05±0.12
Q5	3.41±0.10	3.48±0.11	3.42±0.12	2.81±0.09	3.00±0.11	2.94±0.11	5.91±0.12	5.81±0.14	5.78±0.15
*p* value	0.08	0.12	0.27	**0.00**	**0.00**	**0.01**	0.73	0.67	0.49
**Factor 2: Processed meats and Fish**									
Q1	3.50±0.10	3.45±0.09	3.34±0.10	3.11±0.09	3.06±0.09	2.96±0.09	5.94±0.12	5.92±0.12	5.92±0.12
Q2	3.35±0.10	3.30±0.10	3.24±0.10	3.02±0.09	2.94±0.09	2.84±0.10	5.88±0.12	5.87±0.12	5.83±0.13
Q3	3.16±0.09	3.13±0.10	3.14±0.10	3.08±0.09	3.01±0.09	2.98±0.09	5.78±0.12	5.77±0.12	5.79±0.13
Q4	3.22±0.10	3.22±0.10	3.17±0.10	3.02±0.09	3.03±0.09	2.95±0.09	5.75±0.12	5.75±0.12	5.77±0.13
Q5	3.42±0.10	3.51±0.11	3.48±0.11	2.74±0.09	2.88±0.10	2.83±0.10	6.29±0.12	6.34±0.13	6.26±0.14
*p* value	0.10	0.06	0.19	**0.04**	0.70	0.72	**0.01**	**0.01**	0.10
**Factor 3: Fruits**									
Q1	3.60±0.10	3.62±0.10	3.52±0.10	3.22±0.09	3.24±0.09	3.13±0.09	6.14±0.12	6.17±0.12	6.09±0.12
Q2	3.43±0.10	3.43±0.10	3.31±0.10	3.09±0.09	3.01±0.09	2.91±0.10	5.84±0.12	5.86±0.12	5.73±0.13
Q3	3.18±0.10	3.17±0.10	3.14±0.10	2.91±0.09	2.84±0.09	2.77±0.09	5.68±0.12	5.68±0.12	5.67±0.13
Q4	3.46±0.10	3.44±0.09	3.41±0.10	3.06±0.09	3.03±0.09	2.98±0.09	6.01±0.12	6.01±0.12	6.03±0.12
Q5	2.99±0.10	2.96±0.10	2.98±0.11	2.70±0.09	2.81±0.10	2.78±0.10	5.97±0.12	5.93±0.13	6.04±0.13
*p* value	**<0.001**	**<0.001**	**<0.001**	**<0.001**	**0.01**	**0.05**	0.09	0.08	0.08
**Factor 4: Vegetables and Red Meat**									
Q1	3.53±0.10	3.53±0.09	3.44±0.10	3.15±0.09	3.17±0.09	3.09±0.09	5.96±0.12	5.96±0.12	5.90±0.13
Q2	3.19±0.10	3.14±0.10	3.00±0.10	2.92±0.09	2.81±0.09	2.67±0.09	5.81±0.12	5.81±0.12	5.70±0.13
Q3	3.14±0.10	3.10±0.10	3.10±0.10	2.77±0.09	2.67±0.09	2.64±0.09	5.68±0.12	5.66±0.12	5.69±0.13
Q4	3.30±0.09	3.31±0.09	3.31±0.10	2.95±0.09	2.95±0.09	2.93±0.09	5.99±0.12	6.01±0.12	6.06±0.12
Q5	3.49±0.10	3.53±0.10	3.50±0.10	3.18±0.09	3.32±0.09	3.22±0.10	6.19±0.12	6.21±0.12	6.21±0.13
*p* value	**<0.001**	**<0.001**	**<0.001**	**0.01**	**<0.001**	**<0.001**	**0.04**	**0.03**	**0.02**

^1^All analyses were conducted using analysis of covariance and values are reported as Mean ± Standard Error (SE)

^2^Adjusted for age, sex and total energy

^3^Adjusted for age, sex, total energy, BMI, physical activity, marital status, smoking status, job status, education status, home ownership, diabetes and hypertension

### Dietary patterns and the chance for developing severe mental disorders symptoms

Crude and multivariable-adjusted odds ratios (ORs) and 95% CIs for severe depression, anxiety, and psychological distress symptoms across quintiles of DPs’ scores are presented in Table 6. The analysis revealed that compared with the first quintile, participants in the fifth quintile of “Fruits” dietary pattern had lower odds of severe depression (OR=0.61, 95% CI: 0.45–0.81, p for trend=0.008), anxiety (OR=0.64, 95% CI: 0.50–0.80, p trend=0.001), and stress symptoms (OR=0.45, 95% CI: 0.30–0.68, p for trend=0.001). This association remained significant for depression (OR: 0.63, 95% CI: 0.46–0.87), anxiety (OR=0.64, 95% CI: 0.48–0.84), and stress symptoms (OR=0.46, 95% CI: 0.29–0.74) even after adjustment for all potential confounders in the model; however, the linear trend for the association between this dietary pattern and odds of depression (p=0.057) and psychological distress symptoms (*p*=0.081) became marginally significant in this model. The other dietary patterns were associated with the likelihood of developing depression, anxiety, and psychological distress symptoms neither in crude nor in multi-variable adjusted models.

**Table 6:** The likelihood of developing severe depression, anxiety and stress symptoms according to quintile of dietary food patterns

	Severe Depression			Severe anxiety			Severe Stress		
	Crude	Model 1^**2**^	Model 2^**3**^	Crude	Model 1	Model 2	Crude	Model 1	Model 2
**Factor 1: Sugar and Fats**									
Q1	1	1	1	1	1	1	1	1	1
Q2	0.83 (0.64-1.10)	0.82 (0.64-1.06)	0.88 (0.66-1.16)	0.84 (0.67-1.06)	0.83 (0.66-1.05)	0.90 (0.70-1.17)	1.20 (0.81-1.77)	1.17 (0.80-1.73)	1.30 (0.85-2.00)
Q3	**0.70 (0.54-0.92)**	**0.71 (0.55-0.93)**	**0.72 (0.54-0.97)**	**0.77 (0.60-0.98)**	**0.78 (0.61-0.99)**	0.81 (0.62-1.06)	0.93 (0.62-1.40)	0.94 (0.62-1.42)	1.01 (0.64-1.58)
Q4	**0.66 (0.50-0.87)**	**0.69 (0.52-0.91)**	0.76 (0.56-1.03)	0.80 (0.63-1.01)	0.82 (0.64-1.04)	0.90 (0.69-1.18)	0.81 (0.53-1.24)	0.82 (0.53-1.27)	0.91 (0.56-1.47)
Q5	0.83 (0.64-1.09)	0.97 (0.71-1.32)	1.08 (0.77-1.52)	0.91 (0.72-1.14)	1.00 (0.76-1.32)	1.12 (0.83-1.52)	0.92 (0.61-1.39)	1.00 (0.61-1.63)	1.06 (0.62-1.81)
p for trend	**0.043**	0.164	0.544	0.345	0.530	0.863	0.233	0.460	0.661
**Factor 2: Processed meats and Fish**									
Q1	1	1	1	1	1	1	1	1	1
Q2	0.83 (0.64-1.10)	0.80 (0.61-1.04)	0.83 (0.62-1.11)	0.81 (0.64-1.02)	0.78 (0.62-0.99)	0.81 (0.62-1.05)	1.15 (0.78-1.70)	1.11 (0.75-1.65)	1.04 (0.68-1.59)
Q3	**0.72 (0.56-0.95)**	**0.70 (0.53-0.92)**	0.75 (0.56-1.01)	**0.71 (0.56-0.90)**	**0.69 (0.54-0.88)**	0.78 (0.60-1.01)	0.72 (0.47-1.12)	0.70 (0.45-1.09)	0.66 (0.41-1.05)
Q4	0.85 (0.66-1.11)	0.86 (0.66-1.12)	0.92 (0.69-1.23)	0.79 (0.62-1.00)	0.79 (0.62-1.00)	0.85 (0.66-1.11)	1.08 (0.72-1.60)	1.06 (0.71-1.58)	0.97 (0.63-1.50)
Q5	0.92 (0.71-1.19)	1.06 (0.80-1.40)	1.15 (0.85-1.55)	0.96 (0.77-1.20)	1.06 (0.83-1.35)	1.17 (0.90-1.53)	0.92 (0.61-1.40)	1.02 (0.66-1.58)	0.94 (0.59-1.51)
p for trend	0.609	0.698	0.008	0.664	0.214	0.062	0.607	0.851	0.605
**Factor 3: Fruits**									
Q1	1	1	1	1	1	1	1	1	1
Q2	0.93 (0.72-1.21)	0.93 (0.72-1.21)	0.92 (0.69-1.23)	0.92 (0.73-1.15)	0.92 (0.73-1.16)	0.88 (0.68-1.14)	**0.68 (0.47-0.98)**	**0.66 (0.45-0.97)**	0.65 (0.42-1.00)
Q3	0.78 (0.60-1.01)	0.77 (0.59-1.02)	0.84 (0.62-1.12)	**0.74 (0.59-0.94)**	0.75 (0.59-0.96)	0.78 (0.60-1.01)	**0.50 (0.33-0.75)**	**0.49 (0.32-0.74)**	**0.55 (0.35-0.85)**
Q4	1.02 (0.80-1.31)	1.02 (0.79-1.31)	1.04 (0.79-1.37)	0.90 (0.72-1.14)	0.90 (0.72-1.13)	0.91 (0.71-1.17)	0.69 (0.48-1.00)	**0.68 (0.47-0.99)**	0.74 (0.50-1.11)
Q5	**0.61 (0.45-0.81)**	**0.62 (0.47-0.84)**	**0.63 (0.46-0.87)**	**0.64 (0.50-0.80)**	**0.63 (0.49-0.82)**	**0.64 (0.48-0.84)**	**0.45 (0.30-0.68)**	**0.47 (0.30-0.72)**	**0.46 (0.29-0.74)**
p for trend	**0.008**	**0.019**	0.057	**0.001**	**0.002**	**0.007**	**0.001**	0.839	0.081
**Factor 4: Vegetables and Red Meat**									
Q1	1	1	1	1	1	1	1	1	1
Q2	0.91 (0.70-1.19)	0.87 (0.66-1.13)	0.82 (0.61-1.11)	0.84 (0.66-1.06)	0.82 (0.64-1.04)	0.81 (0.62-1.06)	0.82 (0.54-1.22)	0.77 (0.51-1.16)	0.67 (0.43-1.06)
Q3	0.87 (0.66-1.13)	0.83 (0.63-1.09)	0.87 (0.65-1.17)	0.86 (0.68-1.09)	0.85 (0.67-1.08)	0.90 (0.69-1.17)	0.88 (0.59-1.32)	0.84 (0.56-1.26)	0.82 (0.53-1.28)
Q4	0.90 (0.69-1.16)	0.88 (0.68-1.15)	0.92 (0.69-1.22)	0.94 (0.74-1.18)	0.93 (0.74-1.18)	0.95 (0.74-1.23)	0.90 (0.61-1.33)	0.89 (0.60-1.33)	0.92 (0.60-1.40)
Q5	0.95 (0.73-1.24)	1.00 (0.77-1.31)	1.00 (0.75-1.34)	0.98 (0.77-1.23)	1.01 (0.80-1.28)	1.01 (0.78-1.30)	0.82 (0.55-1.23)	0.85 (0.57-1.29)	0.87 (0.56-1.35)
p for trend	0.705	0.965	0.812	0.831	0.666	0.615	0.493	0.774	0.074

^1^Data are odds ratio (95% CI)

^2^Adjusted for age, sex and energy intake

^3^Adjusted for age, sex, energy intake, BMI, physical activity, marital status, smoking status, job status, education status, home ownership, diabetes and hypertension

## Discussion

In this cross-sectional study, we identified four dietary patterns including “Sugar and Fats”, “Processed Meats and Fish”, “Fruits” and “Vegetables and Red Meat”. We found an inverse association between the “Fruits” pattern and the likelihood of severe depression, anxiety, and psychological distress symptoms, but none of the other dietary patterns were associated with severe mental disorders symptoms.

Psychological disorders impose great socio-economic expenses on individuals and societies and can increase the mortality rate [44]. So, effective strategies to prevent these conditions are necessary [45]. Our results suggested that the “Fruits” dietary pattern, loaded with a high intake of dried fruits, canned fruits, fruit juice, olive and olive oil, hydrogenated fats, and fruits is inversely associated with severe depression, anxiety, and stress. These findings are closely concordant with other reports, in which fruits consumption was shown to be associated with lower odds of psychological disorders [46–48], but several studies have reached no significant association between fruits consumption and psychological disorders [49, 50]. A meta-analysis study on fruit and vegetable consumption and risk of depression was shown that every 100-g increased intake of fruit was associated with a 3 % reduced risk in depression in cohort studies [51]. Several underlying mechanisms could explain the association between the “fruits” dietary pattern and mental health. There are a large number of bioactive compounds such as vitamins, minerals, fiber, antioxidants, flavonoids, and phytochemicals in fruits that may be efficacious in the prevention of mental disorders [52]. The brain is vulnerable to oxidative stress. Oxidative stress, neuroinflammation, and modifications of synaptic molecules are important risk factors of psychological disorders, including depression and anxiety [32]. Antioxidants in fruits such as vitamin C, vitamin E, phenolic compounds, and carotenoids can protect the brain against oxidative, inflammatory, neuronal, and stress-induced damages [53, 54]. Moreover, dietary antioxidants have protective effects against mitochondrial damages, which are common among individuals with psychological disorders [55]. On the other hand, deficiency of some nutrients such as folate might contribute to mental disorders. Folate, as a substance found in fruits, can enhance methylation processes and the regulation of neurotransmitters, such as serotonin, to reduce the risk of depression [48]. In a meta-analysis study, folate has been inversely associated with depression [56]. Olive and olive oil, one of the components of the “Fruits” dietary pattern in our study, may also have an inverse association with psychological disorders. Olive oil produces psychoactive lipid oleamide, which can induce sleep and modulate serotonin receptor-mediated signaling [57]. According to logistic regression, we found that the “Vegetables and Red Meat” dietary pattern, loaded with tomatoes, green leafy vegetables, other vegetables, red meats and fruits had no significant association with depression, anxiety and stress symptoms categories. Previous studies led to inconsistent findings of the relationship between vegetable consumption and psychological health. In line with our research, Pengpid et al. found that vegetable consumption did not significantly decrease the risk of major depression and generalized anxiety disorder [50]. Also, these findings were consistent with a study in Iranian which stated that vegetable consumption was not associated with anxiety and stress [28]. On the other hand, several studies have shown that vegetable consumption has a protective effect against mental disorders [23, 29]. A meta-analysis study on fruit and vegetable consumption and risk of depression was shown that with regard to vegetable consumption, every 100-g increase in intake was associated with 5% reduced odds of depression in cross-sectional studies and 3% reduced risk in cohort studies [51]. One of the justifying reasons that can explain this relationship is that red meats are also loaded in the “Vegetables and Red Meat” pattern, and this might prevent finding the inverse association. Several studies have been found a significant positive association between red meat intake and mental disorders [58, 59].

We found no significant associations between “Processed Meats and Fish” and “Sugar and Fats” dietary patterns and severe mental disorders symptoms. These patterns are loaded with a high intake of sweets and desserts, nuts, snack foods, broth, condiments, sugars and mayonnaise, processed meats, fish, and organ meats. In contrast with our results, a study of Iranian adults, a western dietary pattern characterized by high intakes of sweets and desserts, snacks, chocolate, high-fat dairy products, carbonated drinks, processed meats, mayonnaise, and pickles was associated with increased odds of anxiety in normal-weight participants and depression in men [60]. Jaka et al. concluded that a western dietary pattern characterized by high consumption of meat and liver, processed meats, pizza, salty snacks, chocolates, sugar and sweets, soft drinks, margarine, mayonnaise, and French fries, was associated with increased odds of anxiety in Australian men and women [26]. In line with our results, Nasir et al. found that an unhealthy dietary pattern loaded heavily with high-energy drinks and beverages, fast foods, seasonings, sweets and desserts, snacks, solid fat, pickle, mayonnaise, and high-fat dairy products, did not significantly associate with depression, anxiety, and stress score [61]. It is worth mentioning that the food content of western-type or unhealthy dietary patterns in the different studies, as well as the interactions of various food items in the dietary patterns, might explain these inconsistencies. It should be also mentioned that both healthy and unhealthy food groups were simultaneously loaded in “Processed Meats and Fish” and “Sugar and Fats” dietary patterns and this might explain the non-significant associations found in the present study. The Iranian traditional dietary pattern consists of both healthy and unhealthy food groups including refined grain (white rice and bread), red meat, egg, potato, pickles, hydrogenated fat, sugar, and tea. Several studies have examined the association between Iranian traditional dietary patterns and mental disorders and they have reported inconsistent results and this might be due to the interactions between healthy and unhealthy foods [60, 62].

### Strengths and limitations

The present study has several strengths. The previous investigations from the Middle East were conducted with a limited number of participants while the current study was conducted in a large sample size including both sexes of Iranian adults. Moreover, we adjusted for several important confounders that might affect psychological situations. In addition, the study participants were selected from the general population and this will help the generalizability of our results. This is while the majority of previous investigations were conducted in a specific population, a specific age group, or a particular gender. After all, to the best of our knowledge, it is the first study that reports the relationship between major dietary patterns and severe psychological disorders in a Middle Eastern country; This is while other studies also included those with moderate disorders.

There are several limitations to our study that should be interpreted with caution. First, because of the cross-sectional design, causality cannot be inferred from the current findings; therefore, prospective observational studies like cohort or nested case-control studies are highly necessitated to confirm our results. Although we used a validated FFQ for the assessment of dietary intakes, some degree of measurement error, misclassification, and recall bias might be distorted the results [63]. Moreover, the DASS-21 is not a diagnostic tool and the cut-points for mental health symptom severity were defined according to a previous investigation in Iranians [64]. These may not be comparable to rates of mental health conditions reported in existing study. However, the DASS-21, as a screening tool, has demonstrated a good correlation with tools which have been validated against diagnostic criteria [65]. Besides, the proportions of individuals with severe depression (7.6%), anxiety (10.0%), and stress (3.1%) symptoms were small. The recall bias and misclassification might result in attenuated risk estimates. In addition, the magnitudes of the differences found in Table 5 were extremely small. So, it seems that the differences in depression, anxiety, and stress symptoms across quintiles of dietary patterns are not clinically significant. It should also be noted that although several important confounding variables were adjusted in our study, it is not possible to exclude the effects of residual confounding from unknown or unmeasured factors. It should be considered that we could not assess all psychological determinants of depression, anxiety and stress and adjust them for the associations. The subjective or arbitrary decisions have been made when determining the number of factors to extract and choosing the method of rotation and labeling the main factors. Further cohort studies evaluating the role of other relevant confounders and mediators of this relationship are required to confirm our findings.

## Conclusions

In conclusion, this cross-sectional study demonstrated that individuals who consume a diet higher in dried fruits, canned fruits, fruit juice, olive and olive oil, hydrogenated fats, and fruits have a lower prevalence of severe depression, anxiety, and stress symptoms. Future prospective investigations are required to confirm our findings.

## Supplementary Information

**Additional file 1.**

## Data Availability

The data of the present study will be available for the corresponding author. The data used for the current study are already published in individual papers. The data can be obtained from the corresponding author.

## Abbreviations

DASS-21: Depression, anxiety, and stress scale-21
FFQ: Food frequency questionnaire
IPAQ: International Physical Activity Questionnaire
PCA: Principal component analysis
TAMYZ: TAghzieh-e-Mardome YaZd
YaHS: Yazd Health Survey
YNS: Yazd Nutrition Survey
