# Supplementary material for: The association between major dietary patterns and severe mental disorders symptoms among a large sample of adults living in central Iran: Baseline data of YaHS-TAMYZ cohort study
Source: BMC Public Health. 2022 Jun 4;22:1121. doi: 10.1186/s12889-022-13518-w (PMC9167504; doi:10.1186/s12889-022-13518-w)
Supplement: Supplementary file 1 — Additional file 1. [file 12889_2022_13518_MOESM1_ESM.docx]

**Supplementary Table 1**- Food groups and their corresponding food items used in principal component analysis to derive dietary patterns.

| **Food groups** | **Foods items** |
| --- | --- |
| **Processed meats** | Sausages, Hamburgers |
| **Red meats** | Lamb, Beef, Kebab |
| **Organ meats** | Beef liver, Organ meat (Tongue, Tripe, Head and trotters, Brain, Foot, Abomasum) |
| **Broth** | Broth |
| **Fish** | Canned fish, other fish |
| **Poultry** | Chicken with or without skin (Liver, Heart, Gizzard) |
| **Eggs** | Eggs |
| **Butter** | Butter |
| **Margarine** | Margarine |
| **Vegetable Oil** | Vegetable oils (except for olive oil) |
| **Low fat dairy product** | Low fat milk, Low fat yogurt, Curd |
| **High fat dairy product** | High fat milk, High fat yogurt, Cheese, Cream cheese, Ice-cream, Flavored, chocolate, coffee and honey milk, Cream |
| **Fruit** | Pears, Apricots, Cherries, Apples, Grapes, Bananas, Cantaloupe, Melons, Watermelon, Kiwi, Strawberries, Peaches, Mulberry, Plums, Persimmons, Pomegranates, Figs, Dates, Greengage, Sour cherry, Citrus fruits |
| **Fruit juice** | All types of Natural and artificiel fruit joices |
| **Dried fruit** | Dried Figs, Dried Mulberries, Raisins, dried plums, dried apricots, dried peach, Other dried fruit |
| **Canned fruits** | All types of canned fruit |
| **Tomatoes** | Tomatoes, Tomato paste |
| **Green leafy vegetables** | Spinach, Lettuce |
| **Yellow vegetables** | Carrot |
| **Cruciferous vegetables** | Cucumber; Cabbage; Cauliflower; Brussels sprouts, Turnip |
| **Other vegetables** | Eggplant, Onion, Green beans and peas, Squash, Mushroom, Pepper, Corn, Beet, Garlic |
| **Legumes** | Beans, Peas, Lima Beans, Broad Beans, Lentils, Soy, Split Peas, Mung beans |
| **Potatoes** | Potatoes |
| **French fries** | French fries |
| **Whole grains** | Iranian bread (Sangak), Local bread (Tanoori), Wheat germ, Oatmeal, barley |
| **Refined grains** | White breads (lavash, barbari, baguettes, toast), Noodles, Pasta, Rice |
| **Snacks** | Potato chips, Corn puffs, biscuits and wafers |
| **Nuts** | Peanuts, Almonds, Pistachios, Hazelnuts, Walnuts, Sunflower, pumpkin and watermelon seeds |
| **Mayonnaise** | Mayonnaise sauce |
| **Olive group** | Olives, Olive oil |
| **Hydrogenated fats** | Hydrogenated fats |
| **Sugars** | Jam, Honey, Sugars, Candies, Syrup, Nabat (An Iranian confectionery made of sugar and served by tea), Noql (An Iranian confectionary) |
| **Sweets and desserts** | Chocolates, Cookies, Cakes, Confections, Traditional Sweets (Qottab, Loz), Ardeh (Liquid Sesame), Hlava Shekari (A Sweet Breakfast Food In Iran) |
| **Condiments** | Black pepper, Fried onion, Pomegranate paste, other sauces and pastes |
| **Soft drinks** | Soft drinks |
| **Yoghurt drink** | Dough |
| **Salt** | Salt |
| **Pickles** | Pickles |
| **Tea** | Tea |
| **Coffee** | Coffee and espresso |
| **Pizza** | Pizza |
